# The efficacy and safety of Xianling Gubao capsules in the treatment of knee osteoarthritis: A protocol for a randomized, double-blind, controlled trial

**DOI:** 10.1097/MD.0000000000027086

**Published:** 2021-09-10

**Authors:** Jinfeng Wu, Wenxu Li, Baoqing Ye, Yicun Yao

**Affiliations:** Guangzhou Red Cross Hospital, Guangzhou, Guangdong Province, China.

**Keywords:** blind method, knee osteoarthritis, randomized controlled trials, Xianling Gubao capsule

## Abstract

**Background:**

Knee osteoarthritis (KOA) is a chronic degenerative joint disease, which is the most common type of osteoarthritis. The clinical manifestations are pain, swelling, and dysfunction of the knee joint, which seriously reduces the quality of life of patients and causes a huge social burden. At present, western medicine mainly focuses on symptomatic treatment, such as anti-inflammatory and pain relief, joint cavity injection, joint replacement, etc. The curative effect has certain limitations. Xianling Gubao capsule has some advantages in the treatment of KOA, but it lacks high-quality clinical research to verify it. Therefore, the purpose of this study is to evaluate the efficacy and safety of Xianling Gubao capsule in the treatment of KOA.

**Methods:**

A randomized, double-blind, double-simulation, parallel controlled trial design was used to study the efficacy and safety of Xianling Gubao capsules in the treatment of KOA. The patients were randomly divided into a treatment group and the control group according to 1:1. The treatment group: Xianling Gubao capsule + glucosamine hydrochloride capsule simulation agent treatment; the control group: glucosamine hydrochloride capsule + Xianling Gubao capsule simulation agent treatment. Both groups received standard treatment for 8 weeks and followed up for 30 days. And at the same time, pay attention to its efficacy and safety indicators. Observation indicators include: the western Ontario and McMaster universities osteoarthritis index, hospital for special surgery knee score, liver and kidney function, adverse reactions, etc. Data analysis was performed using SPSS 25.0 software.

**Discussion:**

This study will evaluate the efficacy and safety of Xianling Gubao capsule in the treatment of KOA. The results of this experiment will provide evidence support for Xianling Gubao capsule in the treatment of KOA.

**Trial registration:**

OSF Registration number: DOI 10.17605/OSF.IO/ERM9C

## 1 Introduction

Knee Osteoarthritis (KOA) is the most common degenerative joint disease involving cartilage and surrounding tissues. It is characterized by articular cartilage degeneration, osteophyte formation, and synovial inflammation, which ultimately cause joint pain, stiffness, and dysfunction.^[1,2]^ Among them, pain is the most iconic symptom of KOA, and has the characteristics of progressive joint movement limitation, which brings great inconvenience to the life of middle-aged and elderly people, and seriously affects the quality of life.^[3,4]^ With the increase of population aging, osteoarthritis has become a public health problem that endangers human health around the world.^[5,6]^ In 2010, the global prevalence of symptomatic KOA confirmed by radiology was approximately 3.8%.^[7]^ The incidence of KOA in people over 65 years old is as high as 58% to 68%, and the disability rate can be as high as 53%.^[8]^

The occurrence of KOA is closely related to age, obesity, occupation, trauma, etc.^[9,10]^ There is no unified understanding of the pathogenesis of this disease. Its onset is due to the degenerative changes of articular cartilage under the action of various factors.^[11]^ In addition to joint replacement in Western medicine, conservative treatments often use nonsteroidal antiinflammatory drugs (NSAIDs), glucosamine hydrochloride, sodium hyaluronate intra-articular injections, etc, but they all have different degrees of side effects. Therefore, exploring an effective, convenient, and less adverse reaction therapy is currently the focus of attention.

The disease belongs to the category of “Bi syndrome” in Chinese medicine.^[12]^ In recent years, studies have found that traditional Chinese medicine has advantages in the treatment of KOA, and has fewer adverse reactions.^[13–16]^ However, current related clinical studies mostly focus on the observation of immediate efficacy, and there is a lack of follow-up and clinical efficacy reports related to stability. For this reason, this study compared the curative effect of Xianling Gubao capsule and western medicine in the treatment of KOA.

## 2 Materials and methods

### 2.1 Study design

**Figure 1:**
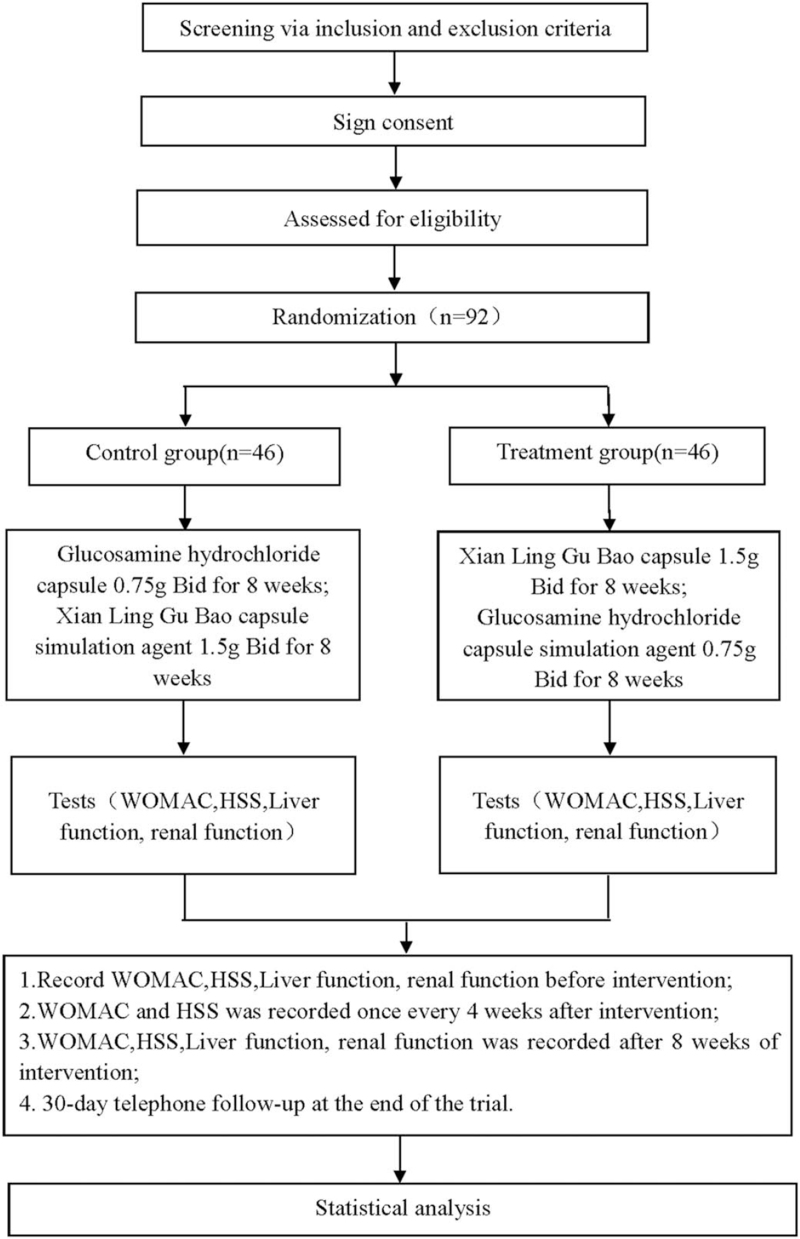
Flow diagram of this study. HSS = Hospital for special surgery knee score, WOMAC = the western Ontario and McMaster universities osteoarthritis index.

This is a randomized, double-blind, double-simulation, parallel controlled trial to study the efficacy and safety of Xianling Gubao capsules in the treatment of KOA. This experiment will follow the comprehensive test report standard. The flowchart is shown in Figure 1.

### 2.2 Ethics and registration

This research protocol complies with the Declaration of Helsinki and was approved by the clinical research ethics committee of our hospital. This experiment has been registered on OSF (registration number: DOI 10.17605/OSF.IO/ERM9C). Before randomization, all patients needed to sign an informed consent form. During the test, they are free to choose whether to continue the test at any time.

### 2.3 Patients

Diagnostic criteria:

(1) Western medicine diagnostic criteria: refer to the KOA diagnostic criteria revised by the American Rheumatism Association^[17]^:
  (a) Knee pain occurs most of the time within 1 month;
  (b) X-ray shows the formation of osteophyte on the joint edge;
  (c) The joint fluid examination is consistent with osteoarthritis;
  (d) Age ≥40 years;
  (e) Morning stiffness ≤30 min; and
  (f) Bone friction sounds when joints are moving.Those who meet the requirements of (a) + (b) or (a) + (c) + (e) + (f) or (a) + (d) + (e) + (f) can be diagnosed as KOA.
(2) Diagnostic criteria of traditional Chinese medicine: refer to the diagnostic criteria of liver and kidney deficiency syndrome of KOA in the “Guiding Principles for Clinical Research of New Chinese Medicines”.^[18]^ Diagnostic criteria for liver and kidney deficiency: Main symptoms: joint pain, waist and knee weakness. Secondary symptoms: poor activity, far-fetched exercise, pale red tongue, thin or thin white coating, slippery or stringy pulse. With the main symptom and more than 2 secondary symptoms, the diagnosis can be confirmed by combining the tongue and pulse.

Inclusion criteria:

(1) those who meet the diagnostic criteria of western medicine for KOA;
(2) those who meet the diagnostic criteria of traditional Chinese medicine for liver and kidney deficiency syndrome;
(3) those who voluntarily receive the corresponding treatment and cooperate; and
(4) those who have signed the informed consent.

Exclusion criteria (a criterion for judging that patients cannot participate in this trial):

(1) Patients with other organ dysfunction;
(2) Patients with other serious diseases such as malignant tumors;
(3) Combined with severe mental illness;
(4) Pregnant or breastfeeding women;
(5) Patients who are allergic to the medications used in this study or have contraindications; and
(6) After explanation, unable to understand the research plan or unwilling to participate.

Exclusion criteria (a criterion for excluding the data of patients participating in the trial):

(1) Severe adverse events or serious complications are not suitable for the next test;
(2) Poor compliance, which affects the judgment of efficacy and safety;
(3) The disease progresses during the treatment process, and the treatment plan needs to be changed; and
(4) For any reason, the subject asked to withdraw from the trial.

### 2.4 Sample size calculation

The main efficacy indicator of this study is the change of visual analogue scale (VAS) score before and after treatment. According to the preliminary clinical trial, the average VAS score before and after treatment of Xianling Gubao capsule is 3.6, and the standard deviation is: 0.6; before and after treatment with glucosamine hydrochloride capsule, the mean of the VAS score is 3.21, and the standard deviation is 1.19. Use PASS 15.0 to estimate the sample size, adopt a superiority design, in which α = 0.025, β = 0.2, test power = 0.8, number of cases in treatment group: number of cases in control group = 1:1, boundary value = −0.4. According to the software calculation, the sample size of the 2 groups was 82 cases. Taking into account the clinical dropout rate of about 10%, a total of 92 cases were finally included. The included patients were numbered according to the order of treatment, and were divided into a treatment group and a control group in a completely random method, with 46 cases in each group.

### 2.5 Study designs

This study will select patients who meet the criteria of this study by recruiting in the hospital. Double-blind and double-simulation were used for the study. The treatment group: Xianling Gubao capsule (Approval Number: Sinopharm Zhunzi Z20025337, Sinopharm Tongjitang (Guizhou) Pharmaceutical Co., Ltd., 0.5g∗40s) oral 1.5 g (3 capsules)/time, 2 times/day, oral glucosamine hydrochloride capsule simulant 0.75 g (1 capsule)/time, 2 times/day, the appearance, smell and taste of the simulant are the same as the original drug, the treatment course is 8 weeks; the control group: glucosamine hydrochloride capsules (Hong Kong Aomei Pharmaceutical Factory, 0.75 g/capsule, production batch number: HC20110009) oral 0.75 g (1 capsule)/time 2 times/day, Xianling Gubao capsule simulant, oral 1.5 g (3 capsules)/time, 2 times/day, the appearance, smell, and taste of the simulant are the same as the original medicine, and the course of treatment is 8 weeks. The 2 groups of patients will receive the same routine care, avoid smoking, alcohol and irritating food during the medication, pay attention to resting, avoid weight bearing on the affected limb, and monitor liver and kidney function. When necessary, the attending doctor can appropriately adjust the plan according to the patient's condition, and all intervention methods will be recorded in detail for the final result analysis.

We arrange for personnel who have nothing to do with clinical trials to complete the blinding and emergency card preparation. Efficacy assessors are not aware of the research group plan, and the data statisticians are not involved in the design and implementation of the study. Before and after treatment, the health status of each patient was evaluated, including efficacy indicators and safety indicators, and all patients were followed up by telephone for 30 days. Follow-up content includes cardiovascular events and rehospitalization.

### 2.6 Evaluation criteria and judgment of curative effect

(1) Observation indicators:
  (a) Using Western Ontario and McMaster Universities Osteoarthritis Index^[19]^ (WOMAC), using V3.1 Mandarin version, through pain, stiffness and joint function. The overall assessment of the joints in 3 aspects, a total of 24 items, each item is recorded by the VAS,^[20]^ the higher the score, the more serious the condition is; and
  (b) Hospital for special surgery knee score^[21]^ evaluates the knee joint function of the patient. The total score of the hospital for special surgery knee score score is 0 to 100 points. The higher the score, the better the knee joint function of the patient.
(2) Adverse reactions: including liver and kidney function abnormalities and any uncomfortable symptoms (such as dizziness, nausea, etc) during treatment.
(3) The clinical efficacy refers to the evaluation criteria for the efficacy of osteoarthritis in the “Guiding principles for clinical research of new traditional Chinese medicine ”^[18]^:
  (a) Significantly effective: the pain symptoms disappear or basically disappear, the joint functions are basically normal, and they can live and work normally, WOMAC points is reduced by ≥70%;
  (b) Effective: Pain basically disappears, joint mobility is slightly limited, life and work ability are improved, 30%≤WOMAC points decrease <70%; and
  (c) Ineffective: Pain and joint function are not significantly improved compared with before treatment, WOMAC points are reduced by <30%. Point reduction rate = [(points before treatment-points after treatment)÷points before treatment] × 100%.

### 2.7 Data collection and management

Before the start of treatment, 4 weeks after treatment, and at the end of 8 weeks of treatment, data were collected according to the evaluation criteria, and each patient was followed up by telephone for 30 days after the end of treatment. It was impossible to collect follow-up information and record the reasons for loss to follow-up. Access to the database is limited to researchers in this research group.

### 2.8 Statistical analysis

In this study, SPSS 25.0 statistical analysis software was used for data analysis. If the measurement data meet the normal distribution, the independent sample *t* test is used between the groups, and the paired sample *t* test is used within the group. The results are represented by ($\bar{x}$ ± *s*); those that do not meet normality, a nonparametric test was used, and the results were expressed in quartiles; the count data were tested by chi-square. *P* < .05 is statistically significant.

## 3 Discussion

At present, Western medicine treatment of KOA mainly uses NSAIDs, glucosamine hydrochloride, glucocorticoids, intra-articular injection of related drugs, or joint replacement to achieve the purpose of alleviating pain and improving joint function. Although it can relieve pain in the short term,^[22]^ the efficacy of these drugs is unstable and all have different degrees of adverse reactions.^[23]^ Among them, the gastrointestinal reaction of NSAIDs often makes patients intolerable and terminates the treatment^[24,25]^; glucosamine hydrochloride can protect the formation of articular cartilage, but for patients with joint cavity effusion, the effect is very small^[26]^; Long-term high-dose application of glucocorticoids can have toxic effects on chondrocytes.^[27]^ In recent years, traditional Chinese medicine has accumulated rich experience in the treatment of bone and joint diseases, and the effect is remarkable, with few adverse reactions. The treatment of KOA by traditional Chinese medicine has become an important direction of clinical research.

Xianling Gubao capsule is currently one of the commonly used Chinese patent medicines in orthopedics and traumatology. It is mainly used for the treatment of osteoarthritis, fractures, osteoporosis, and other orthopedic diseases with good curative effect.^[28]^ Xianling Gubao capsules are composed of: Yin Yang Huo (*Herba Epimedii*), Bu Gu Zhi (*Fructus Psoraleae*), Xu Duan (*Radix Dipsaci*), Zhi Mu (*Rhizoma Anemarrhenae*), Di Huang (sheng) (*Radix Rehmanniae*), Dan Shen (*Radix Salviae Miltiorrhiae*). Yin Yang Huo (*Herba Epimedii*) has the effects of warming liver and kidney, strengthening muscles and bones, and dispelling rheumatism; Bu Gu Zhi (*Fructus Psoraleae*), Xu Duan (*Radix Dipsaci*) replenishing liver and kidney, strengthening muscles and bones; Dan Shen (*Radix Salviae Miltiorrhiae*) dispelling blood stasis and promoting blood circulation; Zhi Mu (*Rhizoma Anemarrhenae*), Di Huang (sheng) (*Radix Rehmanniae*) clearing heat and nourishing yin, nourishing kidney yin; All medicines are used together to replenish liver and kidney, expel wind and dampness, promote blood circulation and clear collaterals. The levels of C-reactive protein, erythrocyte sedimentation rate, and superoxide dismutase in KOA patients increased to varying degrees. Modern pharmacological studies have shown that Yin Yang Huo (*Herba Epimedii*) can stimulate the proliferation of osteoblasts and promote the reconstruction of articular cartilage^[29,30]^; Bu Gu Zhi (*Fructus Psoraleae*) can enhance the activity of bone cells and promote new bone formation.^[31]^ Di Huang (sheng) (*Radix Rehmanniae*) can enhance superoxide dismutase activity and reduce inflammation.^[32]^ Dan Shen (*Radix Salviae Miltiorrhiae*) can resist oxidation and enhance bone metabolism.^[33]^ The active ingredient Tanshinone IIA can promote osteoblast production and inhibit osteoclasts^[34,35]^; combined with the above. This compound can promote the proliferation and differentiation of bone cells in the body, so as to protect the joint function of patients with KOA.

This randomized controlled trial is to verify that Xianling Gubao capsule can improve the clinical symptoms and joint function of patients with KOA. However, this study also has some limitations: due to the short planned follow-up time, we can not understand the impact of long-term efficacy, so we may extend the follow-up time if necessary.

## Author contributions

**Data collection:** Jinfeng Wu and Wenxu Li.

**Funding support:** Jinfeng Wu.

**Investigation:** Baoqing Ye.

**Software operating:** Yicun Yao.

**Study design:** Jinfeng Wu and Wenxu Li.

**Supervision:** Baoqing Ye.

**Writing – original draft:** Jinfeng Wu and Wenxu Li.

**Writing – review and editing:** Jinfeng Wu and Yicun Yao.
